# Whole genome sequencing of *Streptomyces antnestii* sp. nov. with a potency to become an industrial strain

**DOI:** 10.7150/jgen.87156

**Published:** 2024-01-01

**Authors:** Saroja Chhettri, Joseph Sevigny, Céline Pesce, Indrani Sarkar, W.Kelley Thomas, Imen Nouioui, Gargi Sen, Louis S. Tisa, Arnab Sen

**Affiliations:** 1 Department of Botany, University of North Bengal, Raja Rammohanpur, Siliguri-734013, India.; 2 Bioinformatics Facility, University of North Bengal, Raja Rammohanpur, Siliguri-734013, India.; 3 Dept. of Molecular, Cellular and Biomedical Sciences, University of New Hampshire, Durham, NH 03824, USA.; 4 Hubbard Center for Genomic Studies, University of New Hampshire, Durham, NH 03824 USA.; 5 Leibniz Institute DSMZ - German Collection of Microorganisms and Cell Cultures: Braunschweig, Germany.; 6 Midnapore College, Midnapore, West Bengal 721101, India.; 7 Biswa Bangla Genome Center, University of North Bengal, Raja Rammohanpur, Siliguri-734013, India.; 8 Present address: HM Clause, Davis, California, USA.

**Keywords:** *Streptomyces*, Eastern Himalaya, Whole genome sequencing, ANI, MLSA

## Abstract

*Streptomyces* Strain San01 is isolated from the soil of ant-nest found in the tea estate of Darjeeling, India. The morphology, biochemical, as well as the molecular characteristics, proved that San01 belonged to the genus *Streptomyces.* The average nucleotide identity (ANI) value between the genome sequence of the studied strain and its closest phylogenetic neighbors were very low and also could be distinguished from its closest neighbour with broad range of phenotypic data. The draft genome sequence of isolate San01 (NZ_RZYA00000000.1) was estimated to be 9.12 Mbp in size with 71.2% of GC content and it encompasses 39 biosynthetic gene clusters that emphasize the biotechnological potential of this isolate*.*Based on the phenotypic, genetic and genomic data, isolate San01 (=JCM 34633 = NCTC 14543) merits to be recognized as a type strain of a novel species and hereby propose the name *Streptomyces antnestii* sp. nov. Incidentally, this is the first report on *Streptomyces* genomes from Darjeeling, India.

## Introduction

Actinobacteria is a phylum of gram-positive, filamentous bacteria with high GC content that are distributed in a wider range of habitats including terrestrial and aquatic ecosystems 1-3. This phylum is an important player in ecological, agricultural, medicinal, and industrial fields due to its efficient production of secondary metabolites 4-6. Within the Actinobacteria, the genus *Streptomyces* of the family *Streptomycetaceae* 7 and order *Streptomycetales* 8*,* is highly valuable in both medical and industrial aspects as a major bio-resource for antibiotics and other natural products including antivirals, antifungals, antitumoral, anti-parasitic, etc. 9. *Streptomyces* are mainly soil-dwelling saprophytes that have a genome size of 6-12 Mbp 10 and maintain a life cycle with three morphological forms: vegetative hyphae, aerial hyphae, and spores 9. Ecologically, *Streptomyces* can degrade organic compounds, circulate nutrients, and play a pivotal role in maintaining biogeochemical cycles 11.

Darjeeling is part of the sub-Himalayan region in India, is renowned for its tea gardens 12. This region has not been exploited for actinobacterial population studies and no previous report on actinobacterial isolation has been found from this region. Here, we report the draft genome sequence of the *Streptomyces* sp. strain San01 from an ant nest soil found in the tea garden of Darjeeling. The studied strain was further subjected to polyphasic and genomic analyses followed by construction of the 16S and the comparison of the ANI scores for delineation of species. The resultant data confirms its taxonomic status as new species. Therefore, we propose *Streptomyces* sp. San01 as novel species for which the name *Streptomyces antnestii* sp. nov*.* is proposed.

## Materials and Methods

### Isolation and Maintenance of the organism

Soil samples from the ant (*Solenopsis* sp.) nest were collected from Dhajea tea garden situated in Darjeeling (26.9079° N, 88.2230° E), West Bengal, India in April 2016. The collected soil samples were air-dried for three to four days at 28 ℃. Serial dilution of dried soil sample was carried out with sterile saline solution (0.85% NaCl in distilled water) 13 up to 10^-6^ dilution. It was followed by inoculation of 0.1 ml of each dilution onto the sterile growth media by standard spread plate technique. The following growth media were used for isolation: Starch nitrate agar 14, Inorganic Salts, Starch Agar and Nutrient agar media 15. All media were amended with Fluconazole (25µg/ml) and Rifampicin (5µg/ml) before pouring, to minimize the fungal and bacterial contamination 16, 17. The plates were incubated at 30 ºC for five days. Typical colony of actinobacteria was selected and purified by growing on the selective ISP4 medium and incubated for 7 days at 30 °C 18. After two rounds of purification, the isolate was sub-cultured on ISP4 slants and preserved at 4°C for further use 19. Storage of the culture for the long term was carried out at 20% glycerol at -20 °C.

### Morphological, Cultural and Physiological Characterization

Cultural characteristics which include the parameters such as aerial and substrate mycelium formation along with diffusible pigment production were determined according to the International *Streptomyces* Project 20. The isolate San01 was inoculated and incubated for 7 days at 30 °C on various growth media which includes International Streptomyces Project (ISP) Medium 1: Tryptone-yeast extract broth 21, ISP Medium 2: Yeast extract-malt extract agar 22, ISP Medium 3: Oatmeal agar, ISP Medium 4: Inorganic salts-starch agar 24, ISP Medium 5: Glycerol-asparagine agar 23, ISP Medium 6: Peptone-yeast extract iron agar 26, ISP Medium 7: Tyrosine agar 27 and Bennet agar 28 obtained from Hi-Media, Mumbai, India. The gram nature of the strain was confirmed by the gram staining reaction 29. Spore chain morphology of the isolate was studied using ISP4 media by coverslip method 30 following the International *Streptomyces* Project 20. Cellular morphology of the strain was observed using a light microscope and scanning electron microscopy (SEM) 31.

The following biochemical tests were carried out: melanin formation (Shirling and Gottlieb 1966), xanthine, tyrosine and casein decomposition 32, esculin hydrolysis 33, citrate utilization 34, amylase production 35, cellulase production 36, gelatinase production 37, catalase production 38, Lipase production 39, Tween 80 degradation 40, Nitrate reduction test 41, Hydrogen sulphide production 25 and Carbohydrate utilization test 20.

Growth at different pH (5-10), temperature (4°C to 45°C) and NaCl (1 to 10%) tolerance was evaluated on Bennet agar medium. Antibiotic susceptibility was assayed on Bennet Agar (BA) medium by the Kirby-Bauer disc diffusion method 42 using the following antibiotics: Amikacin (30mcg), Ampicillin (10mcg), Amoxycillin (10mcg), Cefadroxil (30mcg), Cefoperazone (75mcg), Ceftazidime (30mcg), Chlorampheniol (30mcg), Ciprofloxacin (5 mcg), Cloxacillin (1 mcg), Cotrimoxazole (25mcg), Erythromycin (15mcg), Gentamicin (10mcg), Nalidixic Acid (10mcg), Netillin (10mcg), Nitrofurantoin (300mcg), Norfloxacin (10mcg), Penicillin (10 units), Tobramycin (10mcg) and Vancomycin (30mcg). Antibiotic discs (Hi-Media Pvt. Ltd., Mumbai, India) impregnated with antibiotics of known amount were placed on the surface of San01 inoculated plates maintaining aseptic condition and incubated at 30 °C for 5 days.

### MALDI-TOF and FAME analysis

Microbial identification of isolate San01 was performed using MALDI-TOF MS 43. Fatty acid Methyl esters (FAME) analysis was carried out for the identification of the bacterial species. The isolate *Streptomyces* sp. San01 cultivated in Inorganic Salt Starch Agar medium (ISP4) for 7 days at 30°C, fatty acids extracted and methylated followed by analysis by Microbial Identification (MIDI) system and the resultant peaks identified using the actino6 database 44.

### Genome Characterization and phylogenetic studies

The genomic DNA of isolate San01 was extracted and purified by CTAB method 30. The 16S rDNA sequence was amplified by Polymerase Chain Reaction (PCR) using actino-specific primers: ACT235F (5'CGC GGC CTA TCA GCT TGT TG3') and ACT 878R (3'CCG TAC TCC CCA GGC GGG G5' 45. The amplified 16S rRNA gene fragments of the *Streptomyces* strain were then sequenced. The sequence similarity was studied through NCBI BLAST program online and similarity-based searches via the ezBioCloud server. Sanger sequencing of the partial 16S rRNA helped us in genus-level identification before proceeding with whole-genome sequencing.

Whole-genome sequencing was performed at the Hubbard Center for Genome Studies (University of New Hampshire, Durham, NH) using Illumina sequencing technology 46. Along with this, a 16S phylogeny was also generated. Average nucleotide identity (ANI) has considered as a potent genome-based criterion for establishing species identity among genetically related micro-organisms 47. The ANI score was calculated by ANI calculator (http://enve-omics.ce.gatech.edu/).

CodonW software (http://codonw.sourceforge.net/) was used for the thorough codon usage analysis. This includes GC composition, Frequency of Optimal codons (Fop), Effective number of codons (Enc), Aromaticity, Hydrophobicity, Codon Adaptation Index (CAI) and Relative Synonymous Codon Usage (RSCU). DAMBE was used for CAI calculation 48. Spearman's Rank correlation coefficient among considered codon usage indices was calculated through SPSS ver 26 software. Genome mining was carried out for the search of biosynthetic gene clusters responsible collectively for bioactive molecule production in the newly sequenced genome using the antiSMASH software version 5.1.0.

## Results and Discussion

### Morphological, Cultural and Physiological Characterization

**Figure 1:**
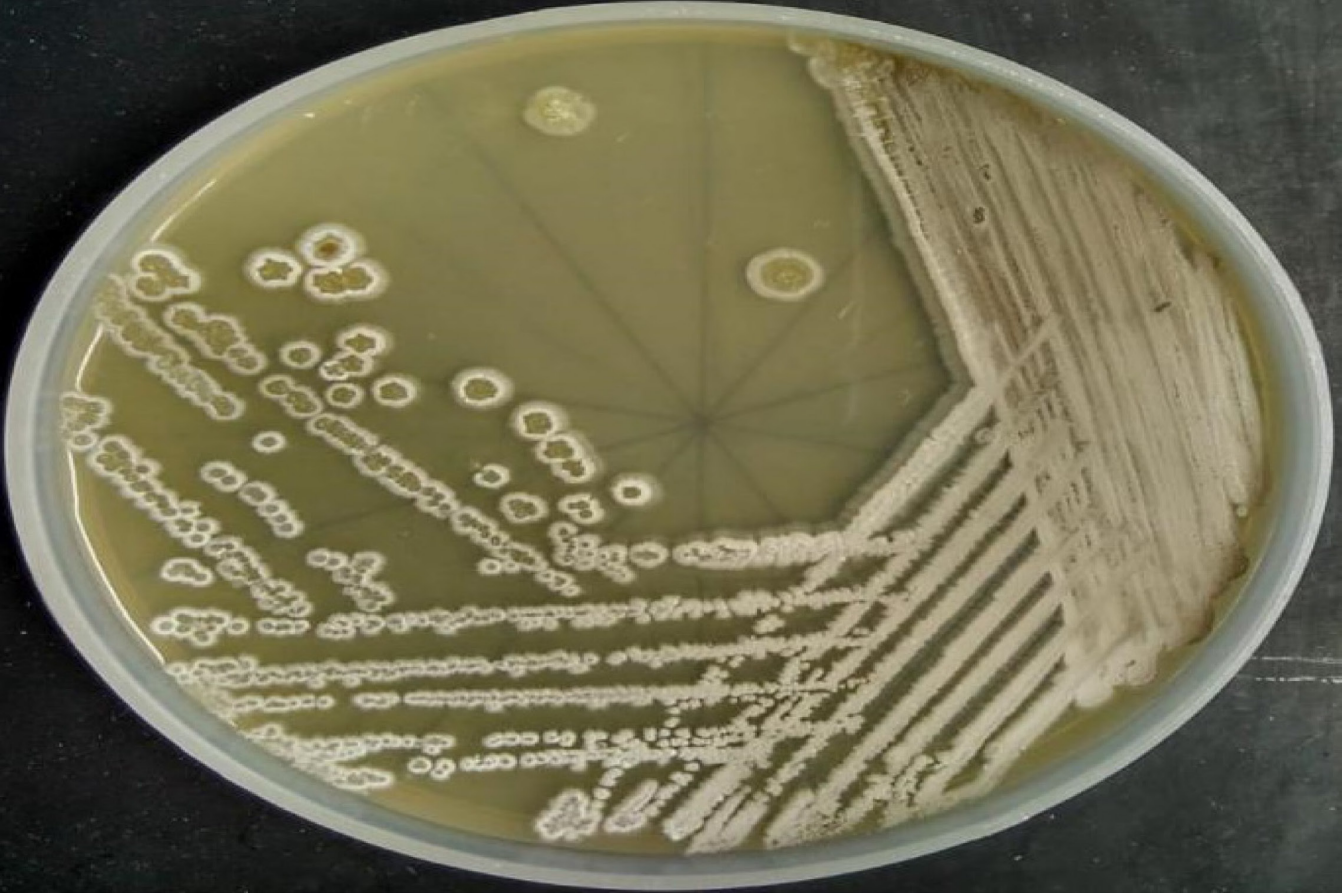
Morphological characteristics of the strain San01- Colony morphology and aerial mycelium of strain San01 after incubation on ISP4 on 7 days.

The colonies of *Streptomyces* sp. San01 are slow-growing, chalky with typical morphology of *Streptomycetes* 49 and sporulated well in ISP4 media (Fig. 1). The strain was gram-positive and filamentous and the spore chains were of flexibilis to looped type (Table 4). The growth of strain at various pH ranges proves the adaptability of the strain at both acidic and alkaline pH. The growth of the strain was observed at the temperature range of 21°C-37°C and showed salt tolerant ability with maximum growth at NaCl concentration of 7% (w/v).

**Figure 2:**
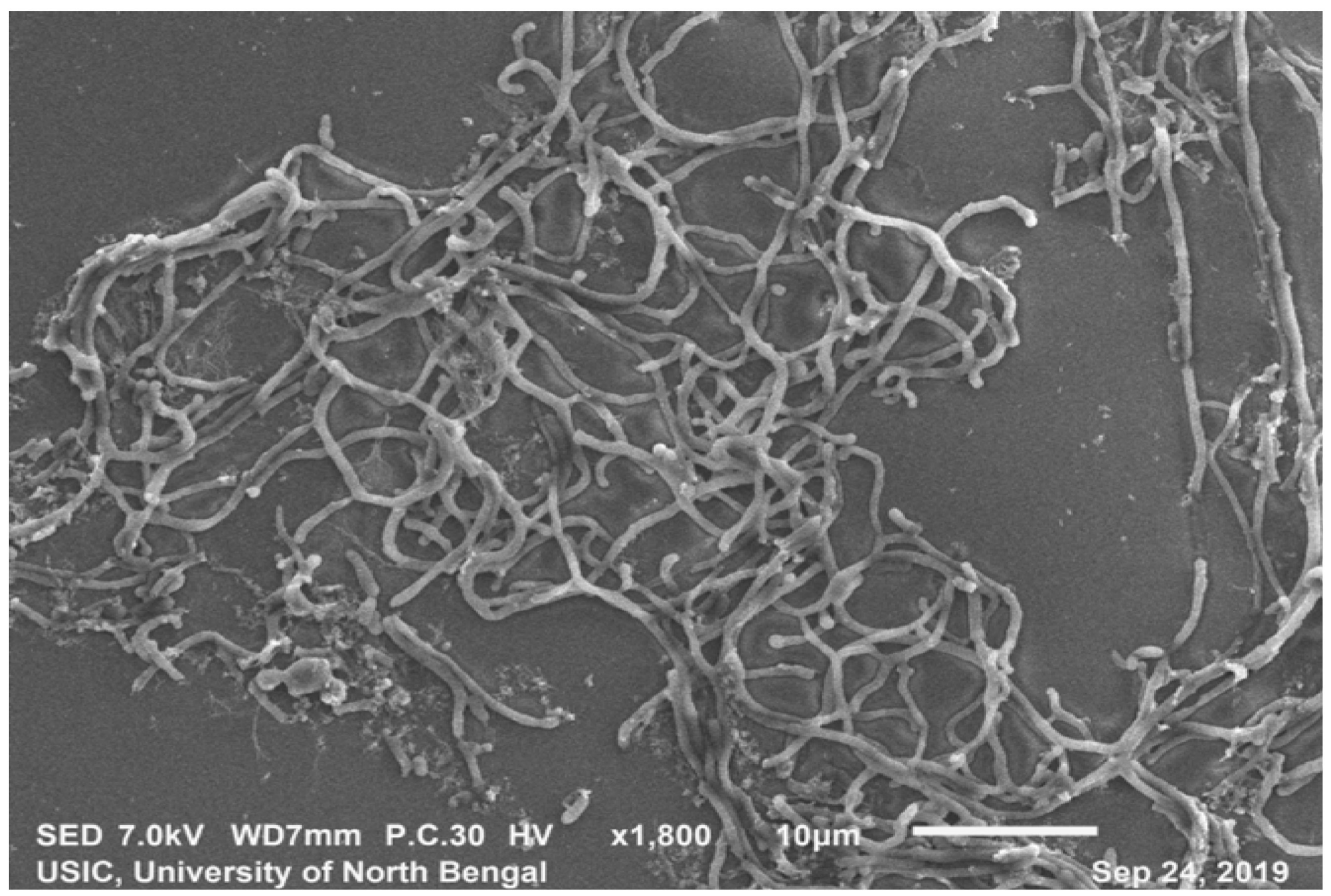
Scanning electron microscopy of San01 after 5 days of incubation in Bennet broth.

SEM analysis revealedthe strain was found to be filamentous with the breadth of the filament in the range of 0.455 µm to 0.645 µm. (Fig. 2). The strain San01 showed variation from* S.aureus* B7319^T^ with Grey substrate mycelium color in contrast with Reddish orange substrate mycelium in the case of *S. aureus* and the spore chain arrangement found to be flexibilis to looped in San01 and Open looped in case of. *S.aureus* B7319^T^.The San01 produced pink pigment on oatmeal agar and *S.aureus* B7319^T^ produced golden pigment. The San01 failed to utilize citrate but *S. aureus* B7319^T^ utilized citrate. The results of the biochemical assays are summarized in Table 1. The isolate showed growth in all the media with good growth observed in Medium: ISP 1, ISP 2, ISP 3, ISP 4, ISP 7 and Bennet agar. The diffusible pink pigment production in ISP 3, ISP 4 and Bennet Agar was observed (Supplementary Table 1). Isolate San01 grew well over a broad range of pH (acidic to alkaline) values and showed optimum growth at 21°C to 37°C temperature range. The isolate was halo-tolerant, exhibiting a maximum growth at 7% (w/v) NaCl. (Supplementary Table 2a, 2b and 2c). The isolate was resistant to Ampicillin (10 mcg), Amoxicillin (10 mcg), Ceftazidime (30mcg), Cloxacillin (1 mcg), Cotrimoxazole (25mcg), Norfloxacin (10mcg), Penicillin (10 units) (Supplementary Table 3). The strains produce industrially significant enzymes Cellulase, Amylase, Gelatinase, and Lipase and could utilize Esculin. The culture isolate showed growth in the presence of Xanthine but could not utilize Citrate and is Catalase negative. Nitrate reduction and Hydrogen sulfide production is absent. It could utilize sugars, fructose, xylose, arabinose, raffinose, rhamnose, mannitol, glucose, sucrose, cellulose, galactose, and lactose but showed no growth or only traces of growth with inositol.

### MALDI-TOF and FAME analysis

MALDI-TOF is one of the analytical techniques with good accuracy for identifying microbes. It revealed the best match with *Streptomyces hirsuitus* with a similarity score 1.717. This score indicates that the isolated strain is a candidate type strain for novel species. FAME analysis using MIDI Sherlock has given a similarity index 0.0 suggests that there is no species in the database that could be identified with isolate San01 (Supplementary Table 4). The major fatty acids of isolate San01 (>15%) was *iso*-C16:0 while the type strain of* S.S.aureus* 41785^T^are*iso*-C_16:0_ and* anteiso*-C_15:0_ as 50 shown in Supplementary Table 4.

### Genome Characterization and phylogenetic studies

The whole genome sequencing of the isolate revealed that the number of contigs in the final draft assembly of *Streptomyces* sp. San01 is 524 with an N50 contig size of 314,067, a total sequence length of 9,124,249 bps, an average coverage of 280x, and a GC content of 71.2%. From the genome sequence of San01 the complete 16S rRNA (1531 bp) gene sequence extracted and were submitted to NCBI with the accession number MZ947234. The whole genome sequences have also been submitted to NCBI with the accession number NZ_RZYA00000000.1.

The annotation of the assembled *Streptomyces* sp. San01 genome was done via the NCBI Prokaryotic Genome Annotation Pipeline (PGAP) 51, which resulted in 7,693 protein-coding genes, 68 tRNA, 10 rRNA (five 5S rRNA, two 16S rRNA and three 23S rRNA), 3 non-coding RNA and 298 pseudogenes. This strain contained 7693 protein-coding genes, 68 tRNA, 10 rRNA (five 5S rRNA, two 16S rRNA and three 23S rRNA), 3 non-coding RNA and 298 pseudogenes. A comparison of the feature annotations produced by the PGAP of *Streptomyces* sp San01 (NZ_RZYA00000000.1.) and the publicly available *Streptomycesaureus strain* NRRL B- 2808 52 (Genebank accession no of LIPQ00000000.1) genome annotations can be seen in Table 2. In the codon usage analysis of the newly sequenced genome of *Streptomyces* sp*.* San 01 it was revealed that 91.8% GC3 count indicated a stringent effect of compositional constrain on this genome. Further, C3 was predominating over G3. The parameters GC, GC3, CAI and Fop were positively correlated with each other with P<0.01 (Table 3).

**Figure 3:**
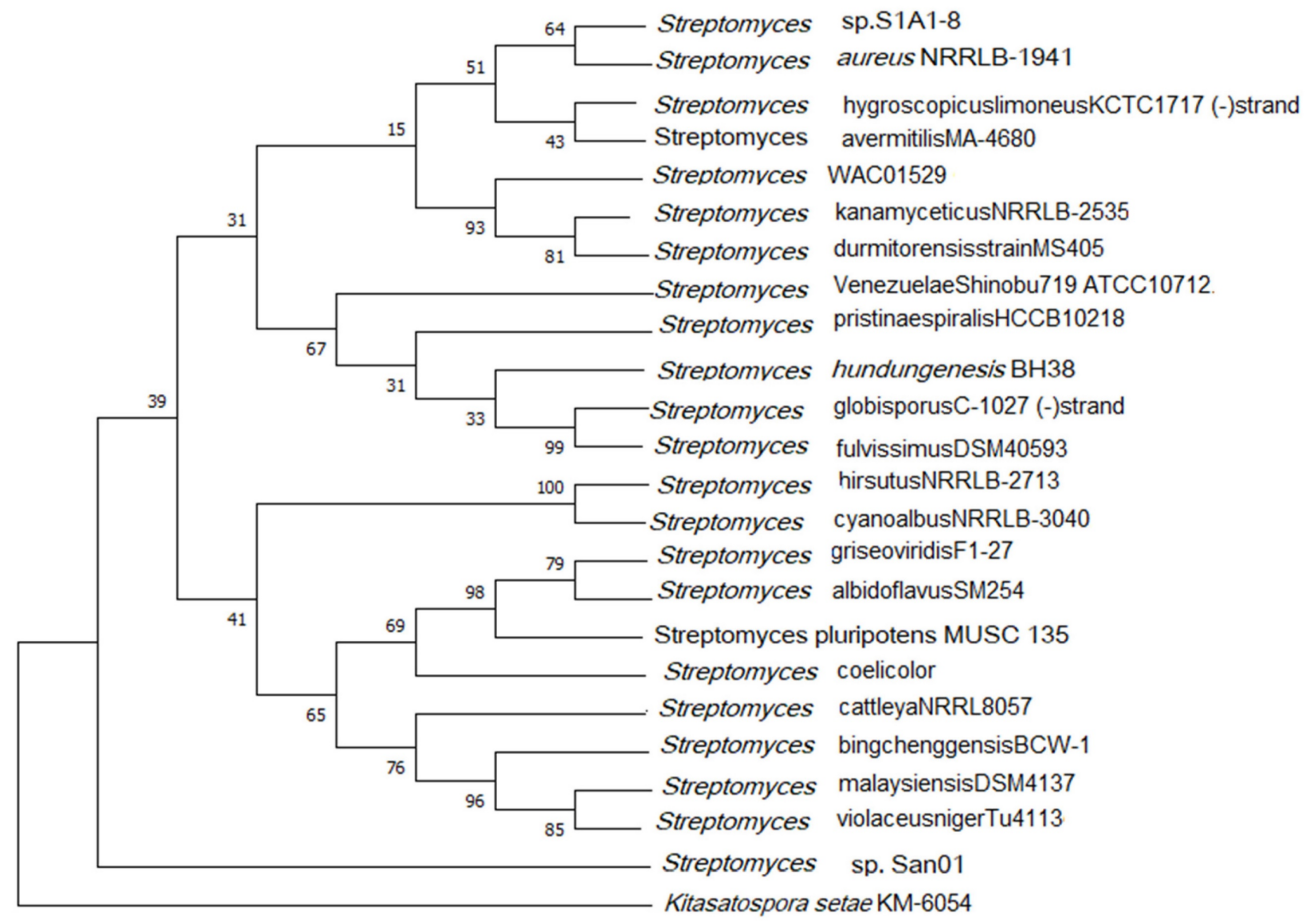
16S rRNA based phylogenetic tree.

The similarity-based searches via ezbiocloud we found that San 01 showed high similarity percentage with *Streptomyces aureus* NRBC100912,* Streptomyces dumitorensis* MS405^T^ and* Streptomyces kanamyceticus* NRBC 13414^T^. ANI is the similarity index between a pair of given genomes and a score of >95% is an indicator that both the genomes are from the same species 53. Thus the ANI scores were calculated between genomes of San 01 and the above-mentioned strains and the ANI scores obtained were 82.50%, 83.67% respectively for *Streptomyces aureus* NRBC100912, and *Streptomyces kanamyceticus* NRBC 13414^T^. However, no genome sequence was found in the database for *Streptomyces dumitorensis* MS405^T^ for ANI calculation. The 16S phylogeny was based on the NJ algorithm with 1000 bootstrap values and the out-group used was *Kitasatospora setae* (Fig 3). Based on this phylogeny it was observed that the studied strain San01 did not cluster with any of the formed groups indicating the uniqueness of this strain.

Bioinformatic analysis revealed a total number of 123 CAZymes related genes for *Streptomyces* sp. San01. CAI (codon adaptation index) values of those genes grouped them under PHX (Potentially highly expressed) genes. This clearly explained the pivotal role of CAZymes related genes among the newly sequenced genome and thereby confirming the role of San01 in the production of important enzymes such as amylase and cellulase. The cluster analysis by antiSMASH software helped in exploring the bioactive potential of the strains 54-56. San01 harbored 32 secondary metabolite biosynthetic gene clusters (BGCs) with more than 70% similarity (Supplementary Figure 1, Supplementary Table 5, and Supplementary Table 6). The presence of numerous BGCs with less similarity to the BGC database provided evidence for the potentiality of the strain to produce diverse secondary metabolites of importance.

The present study is the pioneer work, where the Darjeeling hills are being explored for the isolation of *Streptomyces* species. In this work, the actinobacteria strain isolated from ant nests of tea garden soils is identified as belonging to the genus *Streptomyces* based on morphological, biochemical, microscopic, and 16S rDNA as well as whole genome sequencing studies. The ability of the isolates to produce industrial enzymes like lipase, gelatinase, amylase, and cellulase makes them industrially important. The carbohydrate-activated enzymes (CAZymes) related genes within this genome support the role of the isolate in the production of amylase and cellulase. This emphasizes the importance of the isolate for the exploration of secondary metabolites for industrial applications in the future. The type strain San01 (JCM - 34633 and NCTC -14543) is isolated from the ant nest soil of the tea garden of Darjeeling Hills, India. The GenBank accession number of the assembled draft genome of strain San01 is NZ_RZYA00000000.1.

The Genbank accession number for the 16S rRNA gene sequence of strain San01 is MZ947234.

The Genebank accession number for the whole genome sequence is NZ_RZYA00000000.1.

## Supplementary Material

Supplementary tables.

## Figures and Tables

**Table 1:** Comparison of the biochemical and morphological characteristics of isolate San 01 with the *S.aureus* B7319^ T^ (data taken from Atlan et al. 2000 and Manfio et al. 2003).

Characteristics Studied	Isolate San01	*S.aureus* B7319^T^
Morphology and pigmentation Aerial spore mass colour on oatmeal agar (ISP 3)	Grey	Grey
Pigments on oatmeal agar	Pink	golden
Substrate mycelium colour on oatmeal agar	Grey	Reddish orange
Substrate mycelium colour on Bennett's	Off White to slightly pinkish and greyish	Reddish orange
Substrate mycelium colour on glycerol asparagine	Off White	Reddish orange
Substrate mycelium colour on inorganic salts - starch	grey	Reddish orange
Substrate mycelium colour on yeast extract - malt extract agars.	Off White	Reddish orange
Spore-chain arrangement	flexibilis to looped	Open looped
Melanin pigments production on peptone yeast extract iron agar	-	-
Pigment on inorganic salt starch agar	Pink	Orange yellow
Degradation of:		
Starch	+	ND
Xanthine	+	+
Casein	+	ND
Tyrosine	+	ND
Gelatin	+	ND
Esculin	+	ND
Tween 80	+	ND
tributyrin	+	ND
Nitrate reduction test	-	+
Hydrogen sulphide production	-	ND
Growth on sole carbon source		
Arabinose	+	ND
Dextrin	+	ND
Fructose	+	ND
Meso-Inositol	+	+
Sucrose	+	ND
Xylose	No growth or only traces of growth	ND
Glucose	+	ND
Mannitol	+	+
Rhamnose	+	ND
Cellulose	+	ND
Raffinose	+	ND
Galactose	+	ND
Lactose	+	ND
Utilization of citrate	-	+
Growth at 7% NaCl	+	+
Temperature range for growth	21 to 37	10 to 35

+=positive, - =negative, w =weakly positive,ND=no data available

**Table 2:** Comparison of the genomic data between *Streptomyces* sp. San01 and *S.aureus* NRRL B-2808

Genomic data	*Streptomyces* sp. San01	*Streptomyces aureus* strain NRRL B-2808
Size	9.12 Mbp	7.92 Mb
GC	71.2%	71.8%
Protein coding genes	7,693	6769
RNA	81	79
tRNA	68	72
rRNA	10 rRNA (five 5S rRNA, two 16S rRNA and three 23S rRNA)	4
Noncoding RNA	3	3
Pseudogenes	298	529

**Table 3:** Spearman rank correlation among different codon usage indices. The positive correlation among GC, GC3, CAI and Fop revealed the persistence of compositional constrain on the *Streptomyces* sp. San01. ** indicates P<0.01

	GC	GC3	CAI	ENc	Fop	Aromo
GC	1.00	0.85**	0.87**	-0.75**	0.82**	0.58
GC3		1.00	0.79**	-0.78**	0.85**	0.49
CAI			1.00	-0.72**	0.82**	0.44
ENc				1.00	-0.51	0.32
Fop					1.00	0.42
Aromo						1.00

**Table 4:** Template table that can be used for the purpose of protologue description of a novel species.

Name of a Genus	*Streptomyces*
Name of a Species	*antnestii*
Specific epithet	*antnestii*
Species status	sp.nov
New taxon description	*Streptomyces antnestii* (. *ant nestii* L. gen. n. *antnestii* from ant nest referring to the source of the organism) is aerobic, gram-stain positive, gelatinase-positive actinobacterium which forms an extensively branched substrate mycelium that bears aerial hyphae which is of flexibilis to looped type chains of spores in ISP4 media. The growth of strain at various pH ranges proves the adaptability of the strain at both acidic and alkaline pH. The growth of the strain was observed at temperature range of 21°C-37°C and showed salt tolerant ability with maximum growth at NaCl conc of 7% (w/v). The strains produce industrially significant enzymes cellulase, amylase, gelatinase, lipase and could utilize esculin. The culture isolate showed growth in presence of xanthine but could not utilize citrate and is catalase negative. Nitrate reduction and hydrogen sulfide production is absent. It utilized sugars, fructose, xylose, arabinose, raffinose, rhamnose, mannitol, glucose, sucrose, cellulose, galactose and lactose but showed no growth or only traces of growth with inositol. The predominant fatty acids are anteiso-C_15:0_, C_16:0_, iso-C_16:0,_ anteiso-C_17:1_, C_19:0_.
Origin country	India
Origin region or where the strain belong to	Dhajea, Darjeeling, West Bengal, India
Isolation source	Soil
Date of Sampling	April 2016
Location of site of Sampling	26.9079° N, 88.2230° E
Accession number of 16S rRNA gene	MZ947234
Accession number of Genome	NZ_RZYA00000000.1
Status of Genome	Draft Genome
Size of Genome	9.12 Mbp
G+C %	71.2%
Strain numbers in study	1
Type strain designation	San01
Collection numbers of Strains	JCM 34633, NCTC 14543

## Abbreviations

ISP: International *Streptomyces* Project
BA: Bennet Agar
SEM: Scanning Electron Microscopy
MALDI-TOF MS: Matrix-Assisted Laser Desorption Ionization Mass Spectrometry
FAME: Fatty acid Methyl esters
CAZymes: Carbohydrate- activated enzymes
NRPS: Nonribosomal peptide synthetase
PKS: Polyketide synthase (PKSs)
