# Supplementary material for: Whole genome sequencing of Streptomyces antnestii sp. nov. with a potency to become an industrial strain
Source: J Genomics. 2024 Jan 1;12:6–13. doi: 10.7150/jgen.87156 (PMC10751750; doi:10.7150/jgen.87156)
Supplement: Supplementary file 1 — Supplementary tables. [file jgenv12p0006s1.pdf]

Supplementary table 1: Morphological characterization of San01 using various media:

	**San01**			
	Aerial mycelium	Substrate mycelium	growth	Diffusible Pigment production
Tryptone Yeast Extract Agar(ISP 1)	Off White to grey	Off White to grey	+++	No pigment
Yeast Malt Agar (ISP 2)	Off White	Off White	+++	No pigment
Oatmeal Agar (ISP 3)	grey	grey	+++	Pink pigment
Inorganic Salt Starch Agar(ISP 4)	grey	grey	+++	Pink
Glycerol Asparagine Agar (ISP 5)	Off White	Off White	++	No pigment
Peptone Yeast Extract Iron Agar(ISP 6)	Off White	Off White	++	No pigment
Tyrosine Agar (ISP 7)	grey	Off White	+++	No pigment
Bennet agar	Off white slightly greyish	Off White slightly pinkish greyish	+++	Pink

+++ indicate luxuriant growth,++ indicate good growth,+ indicate weak growth

Supplementary Table 2(a): Growth of San01 at various pH

	pH 5	pH 6	pH 7	pH 8	pH 9	at pH 10
San01	+++	+++	+++	+++	+++	+

+++ indicate luxuriant growth, ++ indicate good growth,+ indicate weak growth

Supplementary Table 2(b): Growth of San01 at various temperature

	4°C	13°C	21°C	28°C	32°C	37°C	40°C	45°C
San01	-	-	+	++	+++	+	-	-

+++ indicate luxuriant growth, ++ indicate good growth,+ indicate weak growth,- indicates no growth

Supplementary Table 2(c): Growth at various % of NaCl**:**

	1% to 3% NaCl	4% NaCl	5 % NaCl	6% NaCl	7 % NaCl	8%NaCl
San01	+++	+++	+++	++	++	+

+++ indicate luxuriant growth, ++ indicate good growth, + indicate weak growth

Supplementary Table 3: Antibiotics Sensitivity and Resistance Profile of *Streptomyces* sp.San01

S.No	Antibiotics	Symbol	Conc in mcg	Sensitivity/Resistance
1	Amikacin	AK	30	Sensitive
2	Ampicillin	AMP	10	Resistant
3	Amoxycillin	AMX	10	Resistant
4	Cefadroxil	CFR	30	Sensitive
5	Cefoperazone	CPZ	75	Sensitive
6	Ceftazidime	CAZ	30	Resistant
7	Ceftriaxone	CTR	30	Sensitive
8	Chlorampheniol	C	30	Sensitive
9	Ciprofloxacin	CIP	5	Sensitive
10	Cloxacillin	COX	1	Resistant
11	Cotrimoxazole	COT	25	Resistant
12	Erythromycin	E	15	Sensitive
13	Gentamicin	GEN	10	Sensitive
14	Nalidixic Acid	NA	10	Sensitive
15	Netillin	NET	10	Sensitive
16	Nitrofurantoin	NIT	300	Sensitive
17	Norfloxacin	NX	10	Resistant
18	Penicillin	P	10 units	Resistant
19	Tobramycin	TOB	10	Sensitive
20	Vancomycin	VA	30	Sensitive

Supplementary Table 4. Comparative Fatty acids profiles of isolate San01with its closely related strain *S. avermitilis* MA-4680^T

Fatty acids	IUPAC Name	*Streptomyces* sp. San01	*S. avermitilis* MA-4680^T
Branched			
iso $C_{14:0}$ (isomyristic acid)	12-methyltridecanoic acid or iso-tetradecanoic acid	2.84	5.1
Iso $C_{15:0}$	13-methyl-tetradecanoic acid	3.04	7.2
anteiso-$C_{15:0}$	(12-methyl-tetradecanoic acid)	9.21	26.1
anteiso-$C_{17:0}$	(14-methyl-hexadecanoicacid)	3.90	15.5
$iso_{16:1}$ H	14-methylpentadecenoic acid	2.37	1.2
iso-$C_{16:0}$	(14-methyl-pentadecanoic acid)	15.70	14.4
Unsaturated Fatty Acids			
anteiso$C_{17:1}$ C	14-methyl-hexadecenoic acid	8.87	-
Saturated Fatty Acids			
$C_{15:0}$	Pentadecanoic Acid	-	6.3
$C_{16:0}$ (Palmitic acid)	Hexadecanoic acid	11.09	15.5
$C_{19:0}$ (Nonadecylic acid)	Nonadecanoic acid	9.93	-

Supplementary Table no 5: Secondary metabolites encoding gene clusters in strain SAn01

Sl.no	MiBiG Cluster	MiBiG Product	Most Similar Known Cluster	Similarity %
1.	BGC0000853	Other	ectoine	93
2.	BGC0000663	Terpene	hopene	91
3.	BGC0002012	Polyketide	julichrome Q3-3	90
4.	BGC0001298	Polyketide	4-Z-annimycin	90

5.	BGC0000658	Terpene	2-methylisoborneol	90
6.	BGC0001083	Terpene + Polyketide:Type III	merochlorin A	90
7.	BGC0000215	Polyketide	curamycin	89
8.	BGC0001598	NRP + Polyketide	foxicins A-D	89
9.	BGC0000271	Polyketide	spore pigment	88
10.	BGC0000171	Polyketide:Modular type I	9-methylstreptimidone	87
11.	BGC0001522	Polyketide	auroramycin	87
12.	BGC0001453	Other	desferrioxamine	86
13.	BGC0000595	RiPP	SCO-2138	86
14.	BGC0001002	NRP + Polyketide	jawsamycin	85
15.	BGC0001979	Polyketide	LL-D49194α1 (LLD)	84
16.	BGC0000941	Other	desferrioxamin B	84
17.	BGC0000238	Polyketide	lactonamycin	83
18.	BGC0000247	Polyketide:Type II + Saccharide:Oligosaccharide	mithramycin	82
19.	BGC0000253	Polyketide:Type II	oviedomycin	79
20.	BGC0000084	Polyketide	laidlomycin	79
21.	BGC0000908	Other	melanin	79
22.	BGC0001080	Other:Phenazine	endophenazine A	77
23.	BGC0000394	NRP	naphthyridinomycin	76
24.	BGC0000270	Polyketide	simocyclinone D8	76
25.	BGC0000171	Polyketide:Modular type I	9-methylstreptimidone	76
26.	BGC0001519	NRP + Polyketide	aurantimycin A	75

27.	BGC0000316	NRP	capreomycin IA	75
28	BGC0000660	Terpene	albaflavenone	72
29	BGC0000503	RiPP:Lanthipeptide	cinnamycin	72
30.	BGC0000336	NRP	daptomycin	72
31.	BGC0001533	Polyketide	borrelidin	72
32.	BGC0000700	Saccharide	istamycin	71

Supplimentary Table 6 : BGCs comparison in San01and *Streptomyces avermitilis* by antiSMASH

	13 common elements in "San01"and "*Streptomyces. avermitilis*":	18 elements included exclusively in "San01":	50 elements included exclusively in "*Streptomyces avermitilis*":
1.	ectoine	2-methylisoborneol	avermitilol
2.	hopene	julichrome Q3-3	filipin
3.	foxicins A-D	4-Z-annimycin	avermectin
4.	spore pigment	merochlorin A	carotenoid
5.	auroramycin	curamycin	oligomycin
6.	desferrioxamine	9-methylstreptimidone	oxazolepoxidomycin A
7.	lactonamycin	SCO-2138	pentalenolactone
8.	laidlomycin	jawsamycin	citrulassin F
9.	melanin	LL-D49194α1 (LLD)	citrulassin D
10.	aurantimycin A	desferrioxamin B	avilamycin A,avilamycin C
11.	albaflavenone	mithramycin	grincamycin
12.	daptomycin	oviedomycin	saprolmycin E
13.	borrelidin	endophenazine A	desotamide
14.		naphthyridinomycin	julichrome Q3-3,julichrome Q3-5
15.		simocyclinone D8	meoabyssomicin, abyssomicin
16.		capreomycin IA	salinomycin
17.		cinnamycin	herboxidiene
18.		istamycin	LL-F28249α
19.			ashimides
20.			JBIR-34, JBIR-35
21.			mannopeptimycin
22.			desferrioxamin B,desferrioxamine E
23.			kirromycin
24.			weishanmycin
25.			herbimycin A

26.			akaeolide
27.			informatipeptin
28.			streptolydigin
29.			diisonitrile antibiotic SF2768
30.			geosmin
31.			siomycin
32.			caniferolide A,caniferolide B,caniferolide C,caniferolide D
33.			rabelomycin,dehydrorabelomycin,fluostatin F,fluostatin G,fluostatin H
34.			flaviolin
35.			mycotrienin I
36.			prejadomycin,rabelomycin,gaudimycin C,gaudimycin D,UWM6,gaudimycin A
37.			WS9326
38.			meilingmycin
39.			formicamycins A-M
40.			α,β-epoxyketone
41.			actinonin
42.			naphthomycin A
43.			cinnabaramide A
44.			flaviolin rhamnoside 3,3'-diflaviolin flaviolin
45.			bottromycin A2
46.			neocarzinostatin
47			polyoxypeptin
48.			nosiheptide
49.			aranciamycin
50			fluostatins M-Q
